# Recent Advances in *Drosophila* Models of Charcot-Marie-Tooth Disease

**DOI:** 10.3390/ijms21197419

**Published:** 2020-10-08

**Authors:** Fukiko Kitani-Morii, Yu-ichi Noto

**Affiliations:** 1 Department of Molecular Pathobiology of Brain Disease, Kyoto Prefectural University of Medicine, Kyoto 6028566, Japan; 2 Department of Neurology, Kyoto Prefectural University of Medicine, Kyoto 6028566, Japan; y-noto@koto.kpu-m.ac.jp

**Keywords:** Charcot-Marie-Tooth disease (CMT), *Drosophila melanogaster*, human disease model, neurodegeneration, peripheral neuropathy

## Abstract

Charcot-Marie-Tooth disease (CMT) is one of the most common inherited peripheral neuropathies. CMT patients typically show slowly progressive muscle weakness and sensory loss in a distal dominant pattern in childhood. The diagnosis of CMT is based on clinical symptoms, electrophysiological examinations, and genetic testing. Advances in genetic testing technology have revealed the genetic heterogeneity of CMT; more than 100 genes containing the disease causative mutations have been identified. Because a single genetic alteration in CMT leads to progressive neurodegeneration, studies of CMT patients and their respective models revealed the genotype-phenotype relationships of targeted genes. Conventionally, rodents and cell lines have often been used to study the pathogenesis of CMT. Recently, *Drosophila* has also attracted attention as a CMT model. In this review, we outline the clinical characteristics of CMT, describe the advantages and disadvantages of using *Drosophila* in CMT studies, and introduce recent advances in CMT research that successfully applied the use of *Drosophila*, in areas such as molecules associated with mitochondria, endosomes/lysosomes, transfer RNA, axonal transport, and glucose metabolism.

## 1. Introduction

### 1.1. Clinical Features of CMT

**Figure 1:**
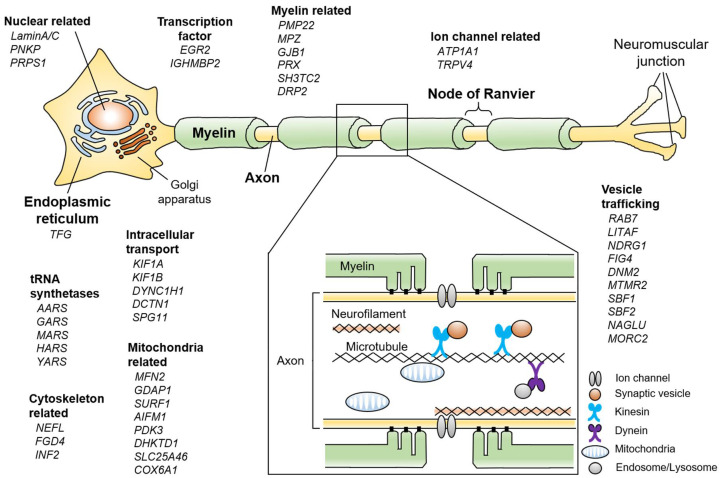
Schematic summary showing various Charcot-Marie-Tooth disease (CMT)-related genes and pathways in the peripheral nerve. If each gene has multiple functions, the most representative one is described. The enlarged box shows a cross-sectional view of the peripheral nerve.

Charcot-Marie-Tooth disease (CMT) is the most common inherited peripheral neuropathy. The average prevalence of CMT is reported to be about 1 in 2,500 people [1]; however, the CMT prevalence rate varies markedly among epidemiological studies due to a large variety of CMT symptoms [2]. At present, the reported prevalence of CMT among Europeans is about 10–30 per 100,000 people, while that in the East Asia is 5.3–10.8 per 100,000 people [3,4,5,6]. The diagnosis of CMT is based on clinical symptoms, electrophysiological studies, genetic testing, and nerve biopsy [7]. CMT is usually juvenile-onset, and typical symptoms are slow, progressive muscle weakness and sensory disturbance in a distal dominant pattern. Patients show clumsiness, foot deformity (such as pes cavus), and gait disturbance. Some patients show additional symptoms such as hearing loss and scoliosis [8]. Interestingly, there are accumulating case reports of cerebral white matter abnormalities mainly in X-linked CMT type 1, and recently, abnormal diffusion-tensor imaging on brain MRI was shown to correlate with clinical disability in various CMT subgroups, suggesting subclinical central nervous system involvement in addition to clinical peripheral neuropathy [9,10,11]. Most patients develop symptoms in childhood, while there are marked individual differences in the severity and progression rate even in those with the same genetic alteration [12]. A nerve conduction study is performed for patients to estimate the background pathology of CMT. A decreased nerve conduction velocity (NCV) (< 38 m/s) indicates CMT1. The main pathological feature of CMT1 is a destruction of the myelin sheath, which is produced by Schwann cells. On the other hand, the presence of decreased compound muscle and sensory action potentials with normal NCV (>38 m/s) indicates CMT2, whose pathological feature is primary axonal damage. The intermediate NCV (30-45 m/s) is associated with the mixed pathology of damaged myelin (demyelination) and axon (axonopathy) [13]. Genetic testing has made marked progress in recent years, and more than 100 genes containing causative mutations have been identified with the widespread use of next-generation sequencing technologies, revealing significant genetic heterogeneity of CMT [2,14,15] (Figure 1). Previous studies showed that about 60% of CMT patients received a definitive diagnosis by genetic testing, and over 90% of genetically diagnosed patients show an alteration in one of the following four genes: *Peripheral Myelin Protein 22-kDa* (*PMP22*), *Gap Junction Beta 1* (*GJB1*), *Myelin Protein Zero* (*MPZ*), and *Mitofusin 2* (*MFN2*) [2,16,17]. However, in the Mediterranean area (e.g., southern Italy and eastern Spain), mutations in *Ganglioside-induced differentiation-associated protein 1* (*GDAP1*) are the third most common genetic diagnosis of CMT after *PMP22* duplication and mutations in *GJB1* [18,19]. No *GDAP1* mutation was identified in a German cohort, suggesting that the geographical area affects the genetic distribution [20]. Nerve biopsy, which was previously the key diagnostic step, is being replaced by genetic testing, but it is still important in atypical cases [7]. Typical findings of nerve biopsy in demyelinating-type CMT are onion bulb formation and a thinned myelin sheath, which are the result of repeated de- and remyelination, and these findings are uniformly observed throughout the nerve. In contrast, axonopathy leads to a decreased number of axons and the disappearance of Schwann cells in the absence of demyelination [21].

### 1.2. Classification of CMT

CMT is clinically divided into subgroups according to the combination of the inheritance pattern and NCV, which helps to estimate the underlying pathology (demyelination or axonopathy) (https://neuromuscular.wustl.edu/) [8] (Table 1). At present, CMT1 is further classified into seven subgroups, from CMT1A to 1G. CMT1A (MIM #118220) is the most frequent subtype of CMT caused by a 1.5-Mb duplication on chromosome 17p11.2 containing the *PMP22* gene, present in about half of all CMT patients [16,17,22]. Mutations within *MPZ* gene are causative for CMT1B (MIM #118200), accounting for around 10% of gene-mutation-identified CMT. CMT1 is the most common subtype of CMT, but CMT1 studies in *Drosophila* are not practical, because *Drosophila* does not have a mature myelin sheath like that of humans.

CMT2, which accounts for 20% of genetically diagnosed CMT patients, shows an autosomal dominant inheritance pattern and decreased nerve action potential amplitudes with normal NCV (which indicates axonopathy) [16,17]. Among many genes in which mutations are reported to cause CMT2, research on the following important genes and their functions has markedly progressed using *Drosophila* models: *MFN2*, *Ganglioside Induced Differentiation Associated Protein 1* (*GDAP1*), *RAB7A, Member RAS Oncogene Family* (*RAB7*), *Glycyl-tRNA Synthetase* (*GARS*), *Alanyl-tRNA Synthetase* (*AARS*), *Methionyl-tRNA Synthetase* (*MARS*), *Histidyl-tRNA synthetase* (*HARS*), and *Sorbitol dehydrogenase* (*SORD*). The proteins MFN2 and GDAP1 are associated with mitochondrial dynamics, RAB7 is associated with the endosomal function, aminoacyl-tRNA transferase (aaRS), which is encoded by *GARS*, *AARS*, *MARS* and *HARS* genes, which are associated with accurate protein translation, and the SORD protein is associated with glucose metabolism (also see Section 2, Section 3, Section 4, Section 5 and Section 6).

The term CMT3 was previously used for infant-onset and the severe CMT subtype. However, CMT3 is no longer used because it proved to be a severe form of early-onset CMT1 or CMT4 by genetic analysis. CMT3, which was historically termed Dejerine–Sottas neuropathy, is now known as the component of the hereditary neuropathies with infantile onset [23,24].

CMT4 is a rare autosomal recessive form of demyelinating CMT with a prevalence of about 1% of genetically identified CMT, although one report suggested a prevalence of 30% in a population with a high rate of consanguineous marriage [16,25]. Unlike CMT1, among various genes in which mutations are reported to cause CMT4, *Factor-induced gene 4* (*FIG4*) of CMT4J (MIM #611228) is actively studied using the *Drosophila* model because FIG4 phospholipid phosphatase has functions including the maintenance of membrane trafficking in both neurons and Schwann cells [26,27,28] (see also Section 3).

All forms of X-linked CMT are designated as CMTX, found in around 10% of all CMT patients [29,30,31,32,33]. CMTX1 (MIM #302800), which is caused by mutations in *GJB1* gene, is the most frequent form and accounts for 90% of CMTX. Among CMTX1 patients, the disease severity and NCV pattern exhibit marked individual differences due to the diversity of mutation sites (more than 250 have been reported) [34]. *GJB1* encodes Connexin-32 that is expressed on the surface of glial cells and forms gap junctions [35]. Recent reports showed that cerebral white matter lesions are sometimes observed in CMTX1 patients [36]. Overall, Connexin-32 is an interesting molecule to investigate the association between axons and myelin sheaths; however, unfortunately, no fly orthologue of human *GJB1* has been identified to date.

### 1.3. Various CMT Models

Until now, various models such as rodents, the zebrafish, fruit fly, yeast, cell lines and induced pluripotent stem cells (iPSCs) have been developed for CMT modeling [37,38,39,40,41,42,43,44,45,46,47,48,49,50,51,52,53,54,55,56,57]. Rodent CMT models have advanced understanding of neuropathy; however, regarding *MFN2* of CMT2A, which is the most frequent subtype of axonal CMT, it used to be difficult to reproduce the phenotype in rodent due to embryonic lethality [58]. In addition, such models are expensive to maintain and are subject to ethical restrictions. Except for the Cartagena Protocol on Biosafety, there are currently no ethical or social restrictions on experiments with *Drosophila* (https://bch.cbd.int/protocol). Regarding the iPSC models, it is also an important tool for analyzing tissues not suitable for biopsy, such as nerves, but there is a limitation whereby the results of in vitro experiments are markedly influenced by the culture conditions. Compared with rodents and iPSCs, the strengths of *Drosophila* as a laboratory animal are as follows: fewer ethical restrictions, a short lifecycle, a large number of genetically homogenous offspring, low maintenance cost and established genetic engineering techniques [59,60]. In the *Drosophila* CMT model, motor deficit in CMT patients is reproduced by a decline in climbing ability during the adult stage and in crawling ability during the larval stage [61]. The locomotive ability of adult flies is assessed by a negative geotaxis assay, first described as climbing activity [62]. Gently tapping a vial containing flies causes them to drop to the bottom, and flies are recorded as they climb up the wall of the vial. The percentage of flies that reach a certain height within a specified time is measured and statistically analyzed. On the other hand, the crawling ability of larvae is used as a method to measure larval locomotive ability [63]. In the larval crawling assay, larvae in the third instar stage are placed on an agar plate and recorded, and the speed of larval migration and distance are statistically analyzed. Although *Drosophila* does not have a mature myelin sheath, the basic structure and physiology of the axons are highly conserved between humans and *Drosophila*, and progress has been made mainly in axonal CMT research. Here, we introduce *Drosophila* models that aided in elucidating the pathophysiology of CMT.

## 2. *Drosophila* CMT Models for Investigating Aberrant Mitochondrial Dynamics

Mitochondria are highly dynamic organelles that are responsible for cell viability. Mitochondria have various functions, such as ATP production, apoptosis, regulation of calcium signaling and reactive oxygen species (ROS) production. Mitochondria continuously repeat fusion and fission to maintain their homeostasis and functions. Mitochondrial fusion promotes diffusion of the matrix content and dilution of oxidized metabolism and damaged mitochondrial DNA, whereas fission is an important process in mitophagy [64]. Mitochondrial fusion and fission are complex processes, and how mitochondrial abnormalities cause disease is not fully understood. However, mutants of following mitochondrial molecules are known to associated with CMT and have been actively studied using *Drosophila*: MFN2, ganglioside-induced differentiation associated protein 1 (GDAP1), and solute carrier family 25 member 46 (SLC25A46).

### 2.1. MFN2

MFN2 is a GTPase localized to the outer mitochondrial membrane and forms a dimer during the process of mitochondrial fusion [65]. Mutations of the *MFN2* gene are causative for CMT2A2 (MIM #609260), which is the most common genotype of inherited axonal-type neuropathy. A previous study showed that mammalian *MFN2* mutations impaired axonal transport of mitochondria [66]. Failure to meet the demand for ATP at the distal axon is one possible cause of neurodegeneration in axonal CMT. Knockdown of *Mitochondrial assembly regulatory factor* (*Marf*), the *Drosophila* homolog of human *MFN2*, also led to phenotypes as follows: (1) motor dysfunction (reduced climbing ability) that rescued with knock-in of the human wild-type *MFN2* but not with *MFN2* with R94Q mutation (one of the most common mutations associated with CMT2A), (2) impaired transport of mitochondria to the distal part of the axon and (3) fragmented and clustered mitochondria [67]. These phenotypes could reproduce key symptoms of patients. The *Marf* knockdown strain also showed fragmented endoplasmic reticulum (ER) cisternae and increased levels of ER stress markers such as X-box binding protein 1 (Xbp1) and binding immunoglobulin protein (BiP), suggesting that ER stress is also involved in the pathogenicity of *Marf* knockdown [67].

Regarding the relationship between mitochondrial morphology and function, Trevisan et al. reported that the neural function was dependent on the capacity of mitochondrial energy production, but was independent of their morphology and distribution [68]. They reported that the single knockdown of *Marf* or *Optic atrophy gene 1* (*Opa1*), each encoding a key molecule for mitochondrial fusion, caused mitochondrial fragmentation, impaired transport of mitochondria, impaired ATP production, and increased lethality in adult flies. Interestingly, double knockdown of genes *Marf* and *Dynamin related protein 1* (*Drp1*), which encoded another GTPase necessary for mitochondrial fission, improved the capacity for ATP production and survival rate but did not rescue aberrant mitochondrial morphology and distribution. On the other hand, double knockdown of *Opa1* and *Drp1* rescued aberrant mitochondrial morphology and distribution, but it did not improve the capacity for ATP production or viability. These results indicate that neuronal cell viability depends on mitochondrial functions rather than the mitochondrial distribution or morphology. In terms of the relationship between the mitochondrial function and morphology, El Fissi et al. reported the diversity of the impaired mitochondrial function and morphology depending on the mutation site in *Marf*. Interestingly, all *Marf* transgenic flies showed reduced climbing ability; flies carrying mutations within the GTPase domain (corresponds to R94Q and T105M in *MFN2*) of *Marf* showed unfused and aggregated mitochondria. On the other hand, flies carrying mutations within the helix bundle 1 domain (corresponds to R364W and L76P in *MFN2*) of *Marf* showed enhanced mitochondrial fusion and giant mitochondria, rescued by overexpression of the fission factor encoding the gene *Drp1* [69]. From these results, not only impaired mitochondrial fusion but also excessive fusion of mitochondria may underlie CMT caused by mutant MFN2, and the diversity of the mutation site in the *MFN2* gene and various functional alterations of MFN2 protein caused by each *MFN2* mutation may lead to the marked individual differences noted in CMT2A patients.

With respect to the development of treatment, Garrido-Maraver et al. showed that folate metabolism-related gene expression was upregulated in *Marf* knockdown *Drosophila*. Although oral folate supplementation did not have a therapeutic effect on this model, overexpression of one gene involved in folate metabolism reduced mortality and ameliorated locomotor deficits in *Marf* knockdown *Drosophila* [70]. These results may help to develop new therapeutic targets. Consequently, Marf/MFN2 is one of the most important molecules in elucidating the effects of mitochondrial abnormality on human disease, and it is expected to continue to be actively studied in *Drosophila*.

### 2.2. GDAP1

Mutations within the *GDAP1* gene are causative for CMT4A (MIM #214400, classified into demyelinating CMT) or CMT2K (MIM #607831, classified into axonal CMT) [71,72]. GDAP1 is a transmembrane protein present in the outer mitochondrial membrane of both neurons and Schwann cells, similar in structure to glutathione S-transferase (GST) but without GST activity [73,74]. GDAP1 is involved in regulation of the mitochondrial morphology and function, but much remains unknown. *GDAP1* gene mutations in patients with an autosomal recessive inheritance pattern were reported to impair mitochondrial fission; however, impaired fusion was also observed in the presence of *GDAP1* mutations in those with an autosomal dominant inheritance pattern [73,74]. López Del Amo et al. showed that knockdown of *dGdap1*, which is a homolog of human *GDAP1*, caused degeneration of the fly’s retina and muscle, and these phenotypes were rescued by human *GDAP1* expression [75]. Both knockdown and overexpression of *dGdap1* resulted in reduced climbing ability. Knockdown of *dGdap1* also resulted in mitochondrial aggregation and large, elongated mitochondria in muscle. On the other hand, overexpression of *dGdap1* resulted in a decrease in the size of mitochondria and cluster formation of mitochondria in the retina. Additionally, with both the overexpression and knockdown of *dGdap1*, early inactivation of the insulin pathway followed by reduced carbohydrate degradation and increased β-oxidation of lipids were observed [75,76]. These *dGdap1*-based results suggest that impaired energy metabolism due to mitochondrial dysfunction may be closely associated with neurodegeneration.

### 2.3. SLC25A46

SLC25A46 is a transmembrane protein that exists in the outer mitochondrial membrane and is considered to be involved in mitochondrial fission. Mutations within the *SLC25A46* gene are causative for hereditary motor and sensory neuropathy (HMSN) type 6B (MIM #616505), and patients exhibit the autosomal recessive inherited form of axonal neuropathy and optic nerve atrophy [50]. The *SLC25A46* knockdown mouse model presented with optic nerve atrophy, axonal degeneration in the peripheral nervous system, giant mitochondria and severe ataxia due to shedding of Purkinje cells in the cerebellum [77]. There are two *SLC25A46* paralogues in *Drosophila, SLC25A46a* [53] and *SLC25A46b* [54]. Previous reports showed that knockdown of each of *SLC25A46a* and SLC25A46b in *Drosophila* similarly led to motor deficit such as reduced crawling and climbing abilities, a shortened synaptic length in the neuromuscular junction (NMJ), decreased ATP production and ROS accumulation [78,79].

It was also reported that human *SLC25A46b* and *Histone deacetylase 1* (*HDAC1*) interacted with each other based on bioinfomatics analysis, and that *Drosophila Histone deacetylase RPD3* (*Rpd3*), a homolog of human *HDAC1*, encoded the protein which regulated the acetylation of histone H4K8 in the *Drosophila SLC25A46b* genomic region. The down-regulation of *Rpd3* expression reduced motor deficits and synaptic morphological abnormalities caused by *dSCL25A46b* knockdown [80]. These findings suggest a novel perspective whereby not only genetic factors, but also epigenetic regulators, may be involved in neurodegeneration due to mitochondrial dysfunction.

## 3. *Drosophila* CMT Models for Investigating Membrane Trafficking Defects

### 3.1. FIG4

Mutations within the *FIG4* gene are causative for CMT4J (MIM #611228) and amyotrophic lateral sclerosis (ALS) type 11 (MIM #612577) [81,82]. ALS is a progressive neurodegenerative disease that selectively damages motor neurons, resulting in motor deficits and fatal respiratory muscle paralysis. *FIG4* encodes a phosphatase present on the surface of late endosomal membranes and it forms a protein complex with Fab1, which is a lipid kinase, and Vac14. This complex regulates the conversion between phosphatidylinositol 3,5-bisphosphate (PI [3,5] P_2_) and phosphatidylinositol 3-phosphate (PI3P), with both being lipids making up membranes that are also responsible for signal transduction [83]. However, recent studies of *Drosophila* revealed a different function of FIG4 other than phosphatase. In *Drosophila*, *dFIG4* is a single homolog for human *FIG4*. Neuron-specific *dFIG4* knockdown *Drosophila* showed ALS-like symptoms such as reduced motor performance, an abnormal morphology of NMJ in motor neurons, and a shortened lifespan. In addition, interestingly, these flies also showed enlarged lysosomes that were partially rescued by expression of the catalytically inactive dFIG4 mutant protein [84,85]. This suggests that dFIG4 functions independently of enzymatic activity to maintain lysosomal membrane homeostasis.

Furthermore, Muraoka et al. and Shimada et al., using genetic screening, showed that *Drosophila* long non-coding RNA (lncRNA) interacted genetically with *dFIG4* and that knockdown of this lncRNA improved phenotypes including motor deficits such as reduced climbing ability and enlarged lysosomes caused by *dFIG4* knockdown [86,87]. Up to now, exome analyses are mainly carried out for the genetic diagnosis of CMT. However, these reports implicate the importance of information on non-protein coding regions in the human genome to elucidate the pathogenesis of CMT. Although the role of these lncRNAs is not yet well-understood, lysosomal membrane metabolism by the FIG4 complex may involve more molecules than previously considered.

### 3.2. RAB7

Mutations within *RAB7* gene are causative for CMT2B (MIM #606071). Rab7 is a small GTPase involved in late endosomal maturation [88,89]. Janssens et al. reported that CMT2B model *Drosophila*, which was expressing a CMT2B-causing *Rab7* mutation in both alleles in all sensory neurons, showed a reduced response to temperature and pain stimuli as well as motor deficit, being similar to the symptoms of CMT2B patients. In the thermotaxis assay, the third instar larvae were placed in the center of a Petri dish with one side warmed to 30℃ and the other maintained at 22℃. Then, less than 10% of control larvae were on 30℃ side, while 17.4% of CMT2B model larvae were on the 30℃ side, suggesting impaired ability of larvae to perceive non-optimal temperatures in this model. In the nociception assay, the authors subjected larvae to touch with a soldering iron maintained at 43℃ as a pain stimulus, which caused a rolling motion in larvae. It took significantly longer to cause a rolling motion in the CMT2B model larvae than in the control, and the proportion of larvae without a rolling motion was also higher in the transgenic CMT2B model larvae. The authors then examined the impact of the CMT2B-causing *Rab7* mutation on vesicle trafficking. Analysis of axonal transport in this transgenic CMT2B model larvae showed a reduced pausing time of RAB7-positive vesicles in the axon [90]. This “reduced pausing time of RAB7-positive vesicles” was confirmed and further investigated in a study using the CMT2B model of *Xenopus* [91]. In this study, a reduced frequency of pausing and an increased velocity of RAB7-positive axonal endosomes were observed in retinal cell axons of *Xenopus* carrying the CMT2B mutation. They also showed that these axonal endosomes transported the ribonucleoprotein particles, and that the CMT2B mutation of *RAB7* reduced the synthesis of axonal mitochondrial outer membrane proteins and altered both the axonal mitochondrial morphology and mitochondrial membrane potential. These results indicate that the RAB7-positive endosome may be the site of local translation in the axon and that such local protein synthesis may be required to maintain the mitochondrial function [91]. These *in vivo* studies of CMT2B-causing *RAB7* mutations will continue to reveal new roles of endosomes and mitochondria in axonal integrity.

## 4. *Drosophila* CMT Models for Investigating Mutant Aminoacyl-tRNA Synthetases

Aminoacyl-tRNA synthetases (aaRS) are enzymes that bind specific amino acids to tRNAs in an ATP-dependent manner. To date, mutants of six types of aaRS have been reported to underlie autosomal dominant axonal or intermediate CMT: Glycyl-tRNA synthetase (GARS) mutants for CMT2D (MIM #601472) and distal hereditary motor neuronopathy 5A (MIM #600287) [92]; Tyrosyl-tRNA synthetase (YARS) mutants for Dominant Intermediate-CMT, Type C (MIM #608323) [93]; Alanyl-tRNA synthetase (AARS) mutants for CMT2N (MIM #613287) [94]; Histidyl-tRNA synthetase (HARS) mutants for CMT2W (MIM #616625) [95]; Lysil-tRNA synthetase (KARS) mutants for CMT recessive intermediate B (CMTRIB, MIM #613641) [96]; Methionyl-tRNA synthetase (MARS) mutants for CMT2U (MIM #616280) [97].

Fly models of CMT caused by mutations in aaRS encoding genes reproduced the clinical phenotypes of CMT patients, such as an impaired motor function, axonal degeneration, muscle denervation and synaptic dysfunction [51,98,99,100,101]; however, notably, the development of motor symptoms did not depend on the enzymatic activity of aaRS in these models. Niehues et al. reported that *Gars*-mutated *Drosophila* with impaired locomotion showed reduced global protein synthesis in peripheral neurons, and this reduced protein synthesis could not be rescued by overexpression of wild-type *Drosophila Gars*. It was suggested that reduced translation may be responsible for the CMT-related phenotypes in *Drosophila*, and that CMT-causing *GARS* mutations might have a toxic gain-of-function effect on protein translation [100]. Furthermore, Bervoets et al. showed that *Drosophila* models with CMT-causing *Yars* mutations exhibited conformational changes in the nuclear YARS protein and over-activation of the transcriptional regulator E2F1. It was suggested that aaRS may have a role as a transcriptional regulator. Regarding the therapeutic approach, both pharmacological prevention of the transfer of mutant YARS protein from the cytoplasm to nucleus and genetic removal of mutant *Yars* from the nucleus improved motor deficits and neural morphological abnormalities in *Drosophila* [102]. Other aaRSs are also expected to have some signal-modulating effect, which may offer new therapeutic targets of CMT.

## 5. *Drosophila* CMT Models for Investigating Impaired Axonal Transport

Axonal transport is essential to maintain axonal homeostasis. Motor proteins transport various cargos such as proteins and mRNA along cytoskeletal filaments. Three large superfamilies of motor protein have been identified: kinesins, dyneins and myosins [103]. Among them, some mutant members of the kinesin and dynein families can cause hereditary neuropathy: Kinesin family member (KIF) 1Bβ for CMT2A1 (MIM #118210) [104], KIF1A for Hereditary Sensory and autonomic neuropathy IIC (MIM #614213) [105], and Dynein, cytoplasmic 1, heavy chain 1 (DYNC1H1) for CMT2O (MIM #614228) [106].

Regarding *KIF1A*, Kern et al. reported that *Drosophila* with the *unc-104* (a synonym of *KIF1A*) hypomorphic mutant showed aberrant apposition of the active zone (AZ) and postsynaptic densities (PSDs) without synaptic retraction, indicating that the protein encoded by *unc-104/KIF1A* might regulate synapse formation and maturation. The authors reported that *unc-104/KIF1A* knockdown resulted in about a 10-fold increase in PSDs unopposed by AZ compared with the wild-type [107]. Further, using the same mutant *Drosophila*, Zhang et al. revealed a decrease in component proteins of synaptic vesicles (such as vesicular glutamate transporter (VGLUT) and cysteine string protein (CSP)) at NMJ and their accumulation in neuronal cell bodies in the presence of *unc-104/KIF1A* knockdown. They also showed that the level of Rab3 protein, which is KIF1A cargo and regulates the exocytosis of synaptic vesicles, was markedly decreased in *unc-104/KIF1A* mutant NMJ. Interestingly, the overexpression of *RAB3* partially rescued aberrant apposition between pre- and postsynaptic structures without the improvement of axonal transport itself, suggesting that the overproduction of Rab3 might enhance feedback from postsynaptic structures, which was markedly impaired in *unc-104/KIF1A* mutant NMJ [108].

Recently, it was shown using an in vitro model that the KIF3 complex bound and transported mRNAs and that the specific sequences of mRNAs increased the selectivity and efficiency of transport [109]. Given the clinical characteristics of CMT such as distal dominancy, impaired synapse maturation caused by impaired intracellular transport may be a significant pathogenesis in CMT.

## 6. *Drosophila* CMT Model for Investigating Mutant Sorbitol Dehydrogenase

Sorbitol dehydrogenase (SORD) is one of the enzymes involved in the polyol pathway and is ubiquitously expressed in mammalian tissues [110]. In the polyol pathway, glucose is converted to sorbitol by aldose reductase and sorbitol is oxidized to fructose by SORD. Under hyperglycemic conditions, accumulations of sorbitol and fructose due to increased polyol pathway flux may contribute to tissue damage [111,112]. In 2020, Cortese et al. reported that biallelic mutations in *SORD* caused inherited neuropathies including CMT and distal hereditary motor neuropathy (MIM #618912) [113]. Notably, in that paper, the pathogenic variant of *SORD*, c.757delG (p.Ala253GlnfsTer27), was shown to be the most frequent cause of the recessive form of inherited neuropathy (frequency of carriers was ~3 per 1,000 individuals). Of 45 patients carrying biallelic mutations in *SORD*, 98% showed distal dominant lower limb weakness and 43% showed sensory deficits (note: 65% of patients showed a reduced sensory action potential, suggesting asymptomatic sensory nerve impairment), and these patients had serum sorbitol levels more than 100 times higher than those of controls. Patient-derived fibroblasts also showed increased intracellular sorbitol. Furthermore, the pathogenicity of biallelic *SORD* mutations was validated by SORD deficiency models of *Drosophila*. *Drosophila* has two functional SORD proteins that are 90% identical to each other, named Sodh1 and Sodh2 [114]. The authors reported that both *Drosophila* with neuron-specific knockdown of *Sodh1* and *Sodh2* and that with homozygous *Sodh2* loss-of-function mutation (*Sodh2^MB01265/MB01265^*) showed motor deficit and progressive neurodegeneration. In detail, these two *Drosophila* models showed reduced climbing ability at a late stage (40 days after eclosion (DAE)) and the loss of photoreceptor terminals in the lamina layer of compound eyes, and the extent of this structural abnormality ameliorated at 10 DAE compared with 2 DAE. In addition, *Sodh2^MB01265/MB01265^* mutant flies exhibited a four-fold increase in sorbitol concentrations in brain homogenates compared with controls. The authors concluded that these results recapitulated the characteristics of patients, such as progressive motor deficit, neurodegeneration, and an elevated sorbitol level. Interestingly, the oral administration of inhibitors of aldose reductase, which converts glucose to sorbitol, decreased sorbitol levels in brain homogenates and improved the climbing ability and abnormal structure of photoreceptor terminals at a late stage in *Sodh2^MB01265/MB01265^* mutant flies. Analysis of the *Drosophila* model, performed concurrently with identification of the novel gene, helped to confirm that *SORD* mutations were pathogenic rather than simply incidental findings.

## 7. Conclusions

The highly conserved neural structure between humans and *Drosophila* facilitates the study of CMT in the *Drosophila* model. Especially, the motor symptoms of CMT patients are well-reproduced by the climbing and crawling assays of *Drosophila*. Studies of CMT using *Drosophila* models only commenced in 2009, and there are still many unstudied genes containing CMT-causing mutations in *Drosophila*. The study of genetic interactions, in which *Drosophila* excels, is an area of research that is difficult using rodent models, and unique developments in this area will promote advancements in CMT research. It is our hope that the use of *Drosophila* will help facilitate such advancements.

## Figures and Tables

**Table 1:** Characteristics of genes in which mutations are reported to cause CMT.

CMT Subtype	Genomic Locus	Gene Symbol	Biological Functions	*Drosophila* Homolog	Phenotype MIM
**CMT1 (Demyelinating, autosomal dominant)**					
CMT1A	17p12	PMP22	Myelin protein	-	118220
CMT1B	1q23	MPZ	Myelin protein	-	118200
CMT1C	16p13.3	LITAF	Regulation of endosomal trafficking	CG13510, CG13559, CG32280	601098
CMT1D	10q21	EGR2	Transcription factor	sr	607678
CMT1E	17p12	PMP22	Myelin protein	-	118300
CMT1F	8p21	NEFL	Neurofilament protein	-	607684
CMT1G	8q21	PMP2	Myelin protein	-	618279
HNPP	17p12	PMP22	Myelin protein	-	162500
**CMT2 (Axonal, autosomal dominant)**					
CMT2A1	1p36	KIF1B	Intracellular transport	unc-104	118210
CMT2A2	1p36	MFN2	Mitochondrial dynamics	Marf	609260
CMT2B	3q21	RAB7	Regulation of vesicular transport	Rab7	600882
CMT2C	12q24	TRPV4	Regulation of calcium ion influx	nan, iav	606071
CMT2D	7p14	GARS	Protein translation	gars, GlyRS	601472
CMT2E	8p21	NEFL	Neurofilament protein	-	607684
CMT2F	7q11	HSPB1	Microtubule regulator and chaperon activity	heat shock protein family B member	606595
CMT2I/J	1q22	MPZ	Myelin protein	-	607736
CMT2K	8q21	GDAP1	Mitochondrial dynamics	dGdap1	607831
CMT2L	12q24	HSPB8	Microtubule regulator and chaperon activity	-	608673
CMT2M	19q13	DNM2	Endocytosis and regulation of cell motility	shi	606482
CMT2N	16q22	AARS	Protein translation	-	613287
CMT2O	14q32	DYNC1H1	Intracellular transport	dynein heavy chain 64C	614228
CMT2P	9q33	LRSAM1	E3 ubiquitin ligase	-	614436
CMT2Q	10p14	DHKTD1	Mitochondrial biogenesis	CG1544	615025
CMT2U	12q13	MARS	Protein translation	mars, MetRS	616280
CMT2V	17q21	NAGLU	Lysosomal enzyme	CG13397	616491
CMT2W	5q31	HARS	Protein translation	hars, HisRS	616625
CMT2Y	9q13	VCP	Regulation of autophagy	TER94	616687
CMT2Z	22q12	MORC2	Fatty acid metabolism	-	616688
CMT2DD	1p13	ATP1A1	Ion channel at Ranvier nodes	-	618036
HMSN-P	3q12	TFG	ER vesicle trafficking	-	604484
**CMT2 (Axonal, autosomal recessive)**					
AR-CMT2A	1q22	LaminA/C	Nuclear membrane protein	Lam	605588
AR-CMT2B	19q13	PNKP	Regulation of phosphorylation of nucleic acids	CG9601	605589
AR-CMT2F	7q11	HSPB1	Microtubule regulator and chaperon activity	heat shock protein family B member	606595
AR-CMT2K	8q21	GDAP1	Mitochondrial dynamics	dGdap1	607831
AR-CMT2P	9q33	LRSAM1	E3 ubiquitin ligase	-	614436
AR-CMT2R	4q31	TRIM2	E3 ubiquitin ligase	-	615490
AR-CMT2S	11q13	IGHMBP2	Transcription factor	CG30094	616155
AR-CMT2T	3q25	MME	Neutral endopeptidase	Nep1, Nep2	617017
AR-CMT2X	15q21	SPG11	Membrane associated	CG13531	616668
AR-CMT2A2B	1p36	MFN2	Mitochondrial dynamics	Marf	617087
HMSN6B	5q22	SLC25A46	Mitochondrial dynamics	Slc25A46b	616505
SCAN3	1p32.3	COA7	Mitochondrial biogenesis	Coa7	618387
HSMN IIC	2q37	KIF1A	Intracellular transport	unc-104	614213
**CMT4 (Demyelinating, Autosomal recessive)**					
CMT4A	8q13-q21.1	GDAP1	Mitochondrial dynamics	dGdap1	214400
CMT4B1	11q22	MTMR2	Regulation of phosphorylation of Phosphatidylinositol	mtm	601382
CMT4B2	11p15	SBF2	Signaling pathway	Sbf	604563
CMT4B3	22q13	SBF1	Signaling pathway	Sbf	615284
CMT4C	5q23-q33	SH3TC2	Myelin maturation	-	601596
CMT4D	8q24	NDRG1	Vesicle transport	MESK2	601455
CMT4E	10q21-q22	EGR2	Transcription factor	-	605253
CMT4F	19q13	PRX	Myelin maturation	-	614895
CMT4G	10q22	HK1	Glucose metabolism	Hex-A	605285
CMT4H	12p11.2	FGD4	Regulation of actin fibers	-	609311
CMT4J	6p21	FIG4	Endo-lysosomal trafficking	dFig4	611228
CMT4K	9q34	SURF1	Mitochondrial biogenesis	Surf1	616684
**X-linked CMT**					
**Dominant**					
CMTX1	Xq13	GJB1	Gap junction formation	-	302800
CMTX3	Xq27	-	-	-	302802
**Semi-dominant**					
CMTX6	Xp22	PDK3	Mitochondrial biogenesis	Pdk	300905
**Recessive**					
CMTX2	Xp22.2	-	-	-	302801
CMTX4	Xq26	AIFM1	Mitochondrial biogenesis	AIF	310490
CMTX5	Xq22	PRPS1	Nucleotide biosynthesis	Prps	311070
**CMT (Intermediate NCV, autosomal dominant)**					
CMT-DIA	10q24	-	-	-	606483
CMT-IB	19p13	DNM2	Regulation of cellular proliferation	-	606482
CMT-DIC	1p35	YARS	Protein translation	yars, TyrRS	608323
CMT-DID	1q22	MPZ	Myelin protein	-	607791
CMT-DIE	14q32	INF2	Regulation of actin fibers	form3	614455
CMT-DIF	3q26	GNB4	Signaling pathway	Gβ13F	615185
CMT-DIG	8p21	NEFL	Neurofilament protein	-	617882
**CMT (Intermediate NCV, autosomal recessive)**					
CMT RIA	8q21.1	GDAP1	Mitochondrial dynamics	dGdap1	608340
CMT RIB	16q23	KARS	Protein translation	kars, LysRS	613641
CMT RIC	1p36	PLEKHG5	Signaling pathway	CG42674	615376
CMT RID	12q24	COX6A1	Mitochondrial biogenesis	levy, COX6AL, CG14077	616039
CMTXI	Xq22	DRP2	Myelin maturation	-	300052

Abbreviations; CMT: Charcot-Marie-Tooth disease; MIM: Mendelian Inheritance in Man; HNPP: Hereditary neuropathy with liability to pressure palsy; HMSN-P: hereditary motor and sensory neuropathy with proximal dominant involvement; ER: Endoplasmic reticulum; SCAN: spinocerebellar ataxia with axonal neuropathy; NCV: nerve conduction velocity.

## Abbreviations

CMT	Charcot-Marie-Tooth disease
HMSN	Hereditary motor and sensory neuropathy
ALS	Amyotrophic lateral sclerosis
NMJ AZ PSDs	Neuromuscular junction Active zone Postsynaptic densities
